# B cell subtypes' kinetics over a 6 monthxs period in CVID patients submitted to influenza and H1N1 immunization

**DOI:** 10.1186/1939-4551-8-S1-A226

**Published:** 2015-04-08

**Authors:** Maira Pedreschi Marques Baldassin, Anete Grumach, Ana Karolina Barreto De Oliveira, Jorge Kalil

**Affiliations:** 1 University of São Paulo Medical School, Brazil; 2 Heart Institute (InCor), Brazil

## Background

Common Variable Immunodeficiency (CVID) is characterized by hypogammaglobulinaemia and impaired specific antibody production, resulting in increased susceptibility to infections, mainly of the respiratory tract. In an attempt to minimize the recurrent episodes of infections, some studies have recommended immunization with inactivated pathogens or subunits, however, experience with vaccine administration in immunodeficient patients is limited.

## Methods

Therefore, we evaluated the kinetics of B cell subtypes and specific antibody production before and 30, 90 and 180 days post Influenza seasonal and Influenza H1N1 vaccination in 35 CVID patients followed at the Division of Clinical Immunology and Allergy of University of São Paulo Medical School and 16 controls. In addition, we assessed the possible beneficial effect of vaccination in CVID patients through application of a symptoms score during a period of one year before and one year after immunization.

## Results

Interestingly, after vaccination, patients presented a significant reduction on the symptoms score, however they did not produce H1N1 and influenza specific antibodies revealing low seroconversion and seroprotection rates when compared to controls. The analysis of B cell kinetics in cell cultures stimulated with Influenza lysate and hemagglutinin peptide following immunization demonstrated a premature expression of switched memory B cell and plasmablasts at 30 days post vaccination which was not maintained for 180 days as observed in controls. Compared to controls, high frequency upon stimulation at 180 days post vaccination was maintained only by marginal zone B cells. Plasmablasts frequency at any time point is strikingly lower than in controls.

## Conclusions

Despite the defect on differentiation and maintenance of memory and plasmablasts with consequent low secretion of specific antibodies, we conclude that CVID B cell subpopulations were capable to recognize and proliferate with Influenza peptides stimulation. It is possible that composing with T cell activation and, marginal zone B cells could be battling to prolong humoral response while linking innate and adaptive immune system reflected by the considerable clinical improvement observed after vaccination.

